# Development of Circadian Oscillators in Neurosphere Cultures during Adult Neurogenesis

**DOI:** 10.1371/journal.pone.0122937

**Published:** 2015-03-31

**Authors:** Astha Malik, Roudabeh J. Jamasbi, Roman V. Kondratov, Michael E. Geusz

**Affiliations:** 1 Department of Biology, Bowling Green State University, Bowling Green, Ohio, United States of America; 2 Department of Public and Allied Health, Bowling Green State University, Bowling Green, Ohio, United States of America; 3 Department of Biological, Geological, and Environmental Sciences, Cleveland State University, Cleveland, Ohio, United States of America; University College London, UNITED KINGDOM

## Abstract

Circadian rhythms are common in many cell types but are reported to be lacking in embryonic stem cells. Recent studies have described possible interactions between the molecular mechanism of circadian clocks and the signaling pathways that regulate stem cell differentiation. Circadian rhythms have not been examined well in neural stem cells and progenitor cells that produce new neurons and glial cells during adult neurogenesis. To evaluate circadian timing abilities of cells undergoing neural differentiation, neurospheres were prepared from the mouse subventricular zone (SVZ), a rich source of adult neural stem cells. Circadian rhythms in *mPer1* gene expression were recorded in individual spheres, and cell types were characterized by confocal immunofluorescence microscopy at early and late developmental stages in vitro. Circadian rhythms were observed in neurospheres induced to differentiate into neurons or glia, and rhythms emerged within 3–4 days as differentiation proceeded, suggesting that the neural stem cell state suppresses the functioning of the circadian clock. Evidence was also provided that neural stem progenitor cells derived from the SVZ of adult mice are self-sufficient clock cells capable of producing a circadian rhythm without input from known circadian pacemakers of the organism. Expression of *mPer1* occurred in high frequency oscillations before circadian rhythms were detected, which may represent a role for this circadian clock gene in the fast cycling of gene expression responsible for early cell differentiation.

## Introduction

Adult neurogenesis produces new neurons from neural stem progenitor cells (NSPCs). This neural plasticity provides interneurons for the mammalian hippocampus, olfactory bulb (OB), and other brain structures throughout life [1]. NSPCs follow a defined progression in cell differentiation that is best understood in the dentate gyrus (DG) of the hippocampus and the subventricular zone (SVZ) near the lateral ventricles [2]. A daily rhythm in cell cycle entry of stem cells has been described in the adult mouse hippocampus [3], indicating that circadian pacemakers may regulate NSPC differentiation. Similarly, circadian gene expression rhythms have been identified in the hippocampus [4] and OB [5], possibly serving to optimize timing of neurogenesis [3] by providing more responsive cells when they are most needed for fine discrimination of sensory information [6]. Adult neurogenesis in many ways follows the behavior of embryonic stem cells, which undergo self-replication and also differentiate into progenitor cells that eventually give rise to various mature cell types [7]. Adult neural stem cells in the SVZ self-renew and produce neurons and glial cells sequentially through several differentiation stages that appear transiently during neurogenesis and have identifiable cell markers [6].

Although in situ hybridization has shown that expression of the core circadian clock gene *mPer2* oscillates in the mouse DG [8], what generates the circadian timing signal is unknown. It remains unclear whether circadian rhythms occur in the heterogenous population of differentiating cells, mature neurons, or the mostly quiescent stem cells. The NSPCs of the DG may contain intrinsic circadian pacemaker capabilities. They may instead be driven by circadian pacemakers located in other cells within these brain regions or clocks elsewhere in the organism [9,10]. Bioluminescence imaging (BLI) of hippocampal explant cultures has revealed circadian rhythms in *mPer2* expression indicating that autonomous circadian clocks are present [4], but the source of the timing signal within this tissue has not been localized further. Daily rhythms in expression of a second clock gene *Per1* in the intact DG are in phase with rhythms of the master circadian clock in the hypothalamic suprachiasmatic nucleus (SCN) [11], suggesting that any NSPC circadian clocks within the DG, or possibly the SVZ, may also be coupled with the circadian timing system.

Circadian rhythms expressed in mouse or rat OB can function independently of the SCN [12]. These oscillations appear to enhance olfactory responsiveness at night [12] and also interact with the SCN’s timing of daily behaviors [13]. Circadian rhythms in *mPer1* and *mPer2* gene expression are present in the mitral and tufted cells of the rat OB and the granule and mitral cells of the mouse OB [14]. Late embryonic neurons from the rat OB express circadian rhythms in action potential frequency [15]. Unlike the DG, progenitor cells of the SVZ produce immature neurons that migrate from the SVZ through the rostral migratory stream (RMS) to become interneurons of the OB [16]. Various sensory stimuli modulate OB neurogenesis. For example, OB granule cells in mice undergo apoptosis at a higher rate following daily scheduled feeding [17], and olfactory cues must be available during a critical window for granule cell maturation between 2 and 4 weeks after neurogenesis in the SVZ [18]. Recently, it has been shown that suckling by pups synchronizes circadian rhythms in the OB of the dam [19].

Embryonic neural stem cells and differentiating stem cells of the adult testis lack detectable circadian rhythms [20,21]. One possible explanation for this absence is the activity of stemness-maintaining genes producing factors that suppress differentiation. These gene regulators may not be compatible with functions of proteins such as mPer1, mPer2, or BMAL1 that serve in the circadian timing mechanism. As reviewed by Gimble et al., [22] studies suggest a close relationship between circadian and stem cell biology through hypoxia-induced transcriptional regulators [23,24], chromatin remodeling enzymes [25,26], the cell cycle inhibitor p21WAF/CIP1 [27], and Wnt signaling [28–30].

To determine when circadian rhythms first appear during adult neurogenesis, in relation to sequential differentiation events, we used a well-characterized paradigm of in vitro adult neurogenesis and applied BLI to monitor *mPer1* gene expression continuously in mouse SVZ neurospheres. These non-adherent clusters of stem cells and progenitor cells in many ways resemble cells undergoing neurogenesis in vivo [31]. Neurospheres were induced to form in suspension cultures containing stem cell medium (SCM) that is devoid of serum but includes epidermal growth factor (EGF) and basic-fibroblast growth factor (FGF2) to suppress differentiation. An exchange with serum-containing medium (SM) or medium containing the serum supplement B27, without added EGF or FGF2, stimulates neurospheres to differentiate and attach as they transform into cell culture monolayers [32]. We describe a correlation between differentiation state of these neural stem cells and their circadian rhythm status.

## Materials and Methods

### Animals

Transgenic *mPer1*::*luc* mice expressing firefly luciferase under control by the *mPer1* gene promoter [33] were bred and maintained in cycles of 12 h light and 12 h dark to entrain their circadian system. Animal procedures were approved by the BGSU Institutional Animal Care and Use Committee and met the requirements of the NRC Guide for Care and Use of Laboratory Animals.

### Neurosphere cultures

Adult male or female C57BL/6 mice (3–5 months old) were euthanized using isoflurane. Brains were removed quickly and coronal slices were made with a Brain Blocker (PA 001 Rat; David Kopf Instruments, Tujunga, CA, USA) and the SVZ region was dissected. The tissue was washed 4–5 times in cold HBSS and then enzymatically digested with papain and DNAseI (Worthington Biochemical, Lakewood, NJ, USA) for 25–30 min at 37°C, followed by 2–3 washes in DMEM with no added growth factors. The tissue was then mechanically triturated and passed through a 40 μm cell sieve (Falcon; BD Biosciences Discovery Labware, Bedford, MA, USA). The cell suspension was washed and centrifuged for 5–6 min 4 times. The supernatant was discarded and the pellet was re-suspended in SCM, which consisted of DMEM with 10 ng/ml FGF2, 20 ng/ml EGF (Life Technologies, Grand Island, NY, USA). Cells were plated at a density of 2.0–2.5 x 10^4^ cells/ml in SCM. After 4–6 days, neurospheres were observed, as described in a previous study [34]. Between 7 and 10 days in culture, neurospheres of at least 50-μm diameter were collected along with the entire contents of the dish and centrifuged for 5 min at room temperature. The pellet was resuspended in 5–7 ml of SCM medium, triturated to form a cell suspension, and plated in fresh SCM, as described for neurosphere cultures [35,36]. Each original dish was passaged into two dishes, and these secondary spheres were used for experiments.

### Stem cell markers and confocal microscopy

Neurospheres were fixed in 100% methanol for 10 minutes and standard immunocytochemistry was applied that was adapted from a previous study of enteric neurospheres [37]. Immunofluorescence staining was used to identify neural stem progenitor cells, neural progenitor cells, neurons and glia. Primary antibodies were used at the following dilutions: chicken anti-Nestin (Aves Labs, Tigard, OR, USA) 1:1000; chicken anti-Dcx (Aves Labs) 1:750; chicken anti-NeuN (Aves Labs) 1:1000; rabbit anti-BetaIII-tubulin (Cell Signaling Technology, Danvers, MA, USA) 1:1000; mouse anti-GFAP (Cell Signaling Technology) 1:1000; rabbit anti-Musashi1 (Msi1, Cell Signaling Technology) 1:1000; rabbit anti-SOX2 (Life Technologies) 1:500. Samples were rinsed after overnight incubation at 4°C, and were incubated for 2 hrs with appropriate Alexa488 and 458 secondary conjugated antibody (Life Technologies). Confocal microscopy of spheres was performed with a DMI3000B inverted microscope (Leica Microsystems, Buffalo Grove, IL, USA) equipped with a Spectra X LED light engine (Lumencore, Beaverton, OR, USA), X-Light spinning-disk confocal unit (CrestOptics, Rome, Italy) and a RoleraThunder cooled CCD camera with back-thinned, back-illuminated, electron-multiplying sensor (Photometrics) with Metamorph software controlling image acquisition and data analysis (Molecular Devices, Sunnyvale, CA, USA). Confocal images were collected with 20X and 40X objectives using standard DAPI, fluorescein, and rhodamine filter wavelengths.

### Neurosphere bioluminescence imaging

Neurospheres maintained in culture dishes containing SCM were transferred manually with 1 ml pipette tips to either SCM, DMEM containing 10% FBS (SM), or DMEM containing the serum supplement B27 at the suggested dilution (Life Technologies). Approximately 10–15 spheres that were 100–200 μm in diameter were moved to a second 35-mm tissue culture dish containing 2 ml medium where they were imaged for up to 8 days to detect any circadian rhythms in bioluminescence. Media contained 100 U/ml penicillin and 100 μg/ml streptomycin. All media used during BLI contained 10 mM Hepes with pH adjusted to 7.2 and bicarbonate levels adjusted for use in room air [38]. To provide synchronization of individual circadian oscillator cells to a common phase of the circadian cycle [39], some of the spheres in SM or SCM were given 20 μM forskolin in 0.01% (v/v) DMSO for 2 hours, which was removed with two SCM exchanges immediately before 0.2 mM luciferin was added and BLI began.

During imaging, the culture dish was covered with a temperature-controlled optical window sealed with silicone grease and maintained at 37°C (Cell MicroControls, Norfolk, VA, USA). Spheres were imaged with a back-thinned, back-illuminated CCD camera cooled to -90°C (CH360; Photometrics, Tucson, AZ, USA) and a 50-mm Nikkor f/1.2 lens (Nikon, Melville, NY, USA) combined with two close-up lenses (+10 and +4 diopter) that were used together. The field of view was 25% of the dish area, and the depth of field was greater than the height of the neurospheres. Neurospheres were illuminated with red LED light when focusing the camera to collect brightfield images and when handling cultures. Luminescence images were captured with 2 x 2 binning and sequential 1-hr exposures over several days for a maximum of 8 days. Images were analyzed using V++ (Photometrics) and ImageJ (NIH) software.

### Data analysis

Bioluminescence images were processed to remove cosmic ray artifacts by keeping the minimum value at each pixel when comparing every two frames in the time series. A single region-of-interest (ROI) was drawn over each sphere at each frame in the time series. The ROI was moved when needed to correct for any movement of the sphere, but it remained the same size and shape. Spheres that produced a detectable signal for at least 5 days of imaging were analyzed. The first 12 hours of imaging was excluded to eliminate the initial surge in bioluminescence after luciferin was added. Detrending the BLI data was done by 24-point running average subtraction as described previously [40]. A five-point running average was then applied, and the times when peaks occurred were measured using the Peak Analyzer routine in OriginLab 9.0 software (OriginLab, Wheeling, IL, USA). As described previously [38], we used a similar criterion to remove the effects from transient or damping signals to find the peak, which is the highest time point between a rising and a falling phase. Peaks, when identified by Peak Analyzer, were accepted only if the amplitude was greater than or equal to 30% of the amplitude of the peak occurring before and the one during the next peak following the cycle. Amplitude was calculated as the difference between the peak and the trough, which was the previous minimum after the last falling phase. Using the peak phase of each circadian cycle, Rayleigh’s test for uniformity was performed using Oriana circular statistics (Kovach Computing Services, Pentraeth, Wales, UK) to determine whether the phases of circadian rhythms were significantly clustered.

Confocal fluorescence images were collected in a Z-series, and frames that were approximately one third of the distance into the sphere were deconvolved with Autoquant 3D deconvolution. The percentage of neural stem cells was then measured using the Metamorph Multi-Wavelength Cell Scoring routine after background intensity was subtracted based on the average intensity measurements from controls in which primary antibody was omitted. Threshold for detection was 50% of the maximum pixel intensity. Other data set means were compared using Tukey’s multiple comparison test, Chi-square analysis, Mann-Whitney U test, and one-way analysis of variance (ANOVA) followed by Scheffe post hoc test (p< 0.05). Linear correlation was performed with OriginLab.

## Results

### Circadian rhythms are rare in neurospheres maintained in stem cell medium

**Fig 1:**
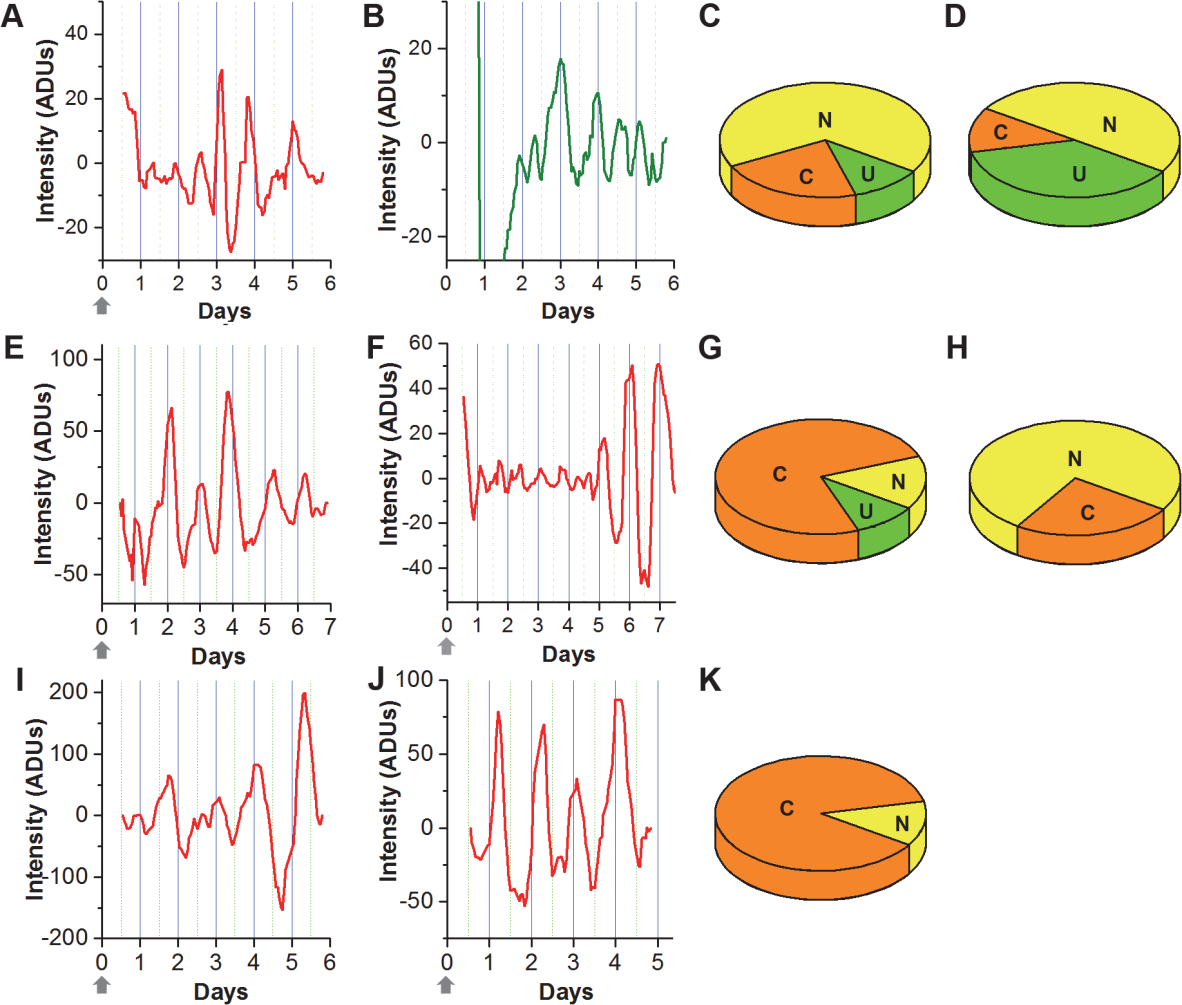
Changes in *mPer1* expression from ultradian to circadian during neurosphere cell differentiation. Bioluminescence was recorded from individual spheres that were first treated with forskolin and then maintained in SCM (**A**) or stimulated to differentiate in SM (**E, F**) or B27 medium (**I**, **J**). Bioluminescence was also recorded from spheres that were not treated with forskolin before maintenance in SCM (**B**). Shown is the 5-point running-average of detrended data as analog-to-digital units of the camera (ADUs). The proportion of spheres that were ultradian (U), circadian (C) and non-rhythmic (N) after 4 days are shown with or without forskolin treatment for spheres in SCM (**C**, **D**), SM (**G**, **H**) and B27 medium (**K**). Arrows indicate when the 2-hr forskolin pulse ended.

To identify the status of circadian rhythms in SCM, SVZ neurospheres were prepared from *mPer1*::*luc* mice [33] and imaged in SCM for 6–7 days. The first 3 and last 3–4 days (early and late components) as well as the entire time series were analyzed. Measurements were made from spheres in four dishes. This procedure was repeated using spheres in SCM without the forskolin pulse (two dishes). Average bioluminescence intensity recorded over time from each sphere was characterized as either circadian (19–29 hrs, Fig 1A), ultradian (<19 hrs, Fig 1B), or non-rhythmic (>29 hrs or no significant oscillation) based on the strongest frequency component of a Lomb-Scargle spectral analysis after detrending the signal as described previously [39]. Only 2 of 9 were circadian in the forskolin-treated SCM group, and these oscillations lasted for only one cycle (Fig 1A and 1C). One of 8 spheres in the non-forskolin group was circadian (Fig 1D). When imaged in SCM, irrespective of forskolin treatment, spheres showed primarily ultradian *mPer1* expression (chi-square test, χ^2^_0.05,15_ = 24.996, p<0.05 followed by a Tukey multiple comparison post hoc test q_∞ 0.05,15_ = 4.38, p<0.05). Many spheres had low-frequency oscillations that were beyond the circadian range and were not of further interest in this study.

### Circadian rhythms in *mPer1* gene expression emerge in neurospheres during differentiation in serum medium or B27 medium

Neurospheres were isolated from culture in SCM and moved to a second culture dish containing SM to induce cell differentiation. Neurospheres were imaged with or without forskolin synchronization. Analysis of the late component of the time series showed that 75% of neurospheres were circadian (15 of 20) and 10% were ultradian (2 of 20) in SM after forskolin synchronization (Fig 1E–1G), whereas 25% were circadian (2 of 8) and no ultradian rhythms were detected (0 of 8) in the SM group not treated with forskolin (Fig 1H). Significantly more circadian rhythms were present in SM than in SCM, with or without forskolin synchronization (Mann Whitney U test, p = 0.02; χ^2^_0.05, 5_ = 11.07, p<0.05, q_∞ 0.05,5_ = 3.69, p<0.05). The proportion of spheres expressing ultradian rhythms after forskolin treatment was not significantly different in SM, SCM, or B27 medium (p>0.05).

Average periods of circadian spheres, based on peak-to-peak intervals, are shown in Table 1. When the periods at the first and third cycles were compared to evaluate the stability of rhythms over time there was no significant difference between SM and B27 spheres (paired t-test, p>0.05). Both groups had been treated with forskolin. A linear regression was also used to identify any effect of time in culture on period for these two groups, and there was no significant change in either direction (SM: r = 0.019, R^2^ = 0.011; B27: r = 0.062, R^2^ = 0.005). Also, there was no significant correlation between amplitude and period when all spheres were analyzed (r = -0.124, R^2^ = -0.025, n = 27) or when the SM and B27 groups were analyzed individually.

**Table 1:** Summary of neurosphere circadian periods.

**Culture condition**	**Spheres tested**	**Spheres circadian**	**Average period ±SD (hours)**	**Mean amplitude ±SD (ADUs)**
SCM with forskolin treatment	9	2	23.5 ±6.3	26.21 ±13.6
SCM without forskolin treatment	8	1	22	38.13 ±26.2
SM with forskolin treatment	20	15	24 ±3.0	103.6 ±101.1
SM without forskolin treatment	8	2	21 ±2.8	61.5 ±42.0
B27 medium with forskolin treatment	8	7	21.71 ±3.3	84.52 ±27.5

Spheres were imaged in SCM and SM with or without forskolin synchronization or in B27 medium. Mean amplitude of spheres that were circadian by Lomb-Scargle analysis was measured on the 2^nd^ cycle during the last 3–4 days of imaging (late). Periods were determined from peak-to-peak intervals of all cycles.

As a second way to induce differentiation, two dishes of SCM-grown spheres were given forskolin treatment and then imaged in B27 medium. Analysis of the late component showed that 87.5% of the neuropheres were circadian (Fig 1I–1K), and there was no significant difference in the number of circadian spheres between the B27 and SM forskolin-treated groups (t-test, p = 0.315). No ultradian rhythms were detected in the B27 forskolin-treated group. Compared to SCM, circadian rhythms were more frequently observed in forskolin-treated spheres imaged in B27 medium (p = 0.050; χ^2^_0.05,5_ = 23.12, p< 0.05, q_∞ 0.05,5_ = 3.76, p< 0.05).

**Fig 2:**
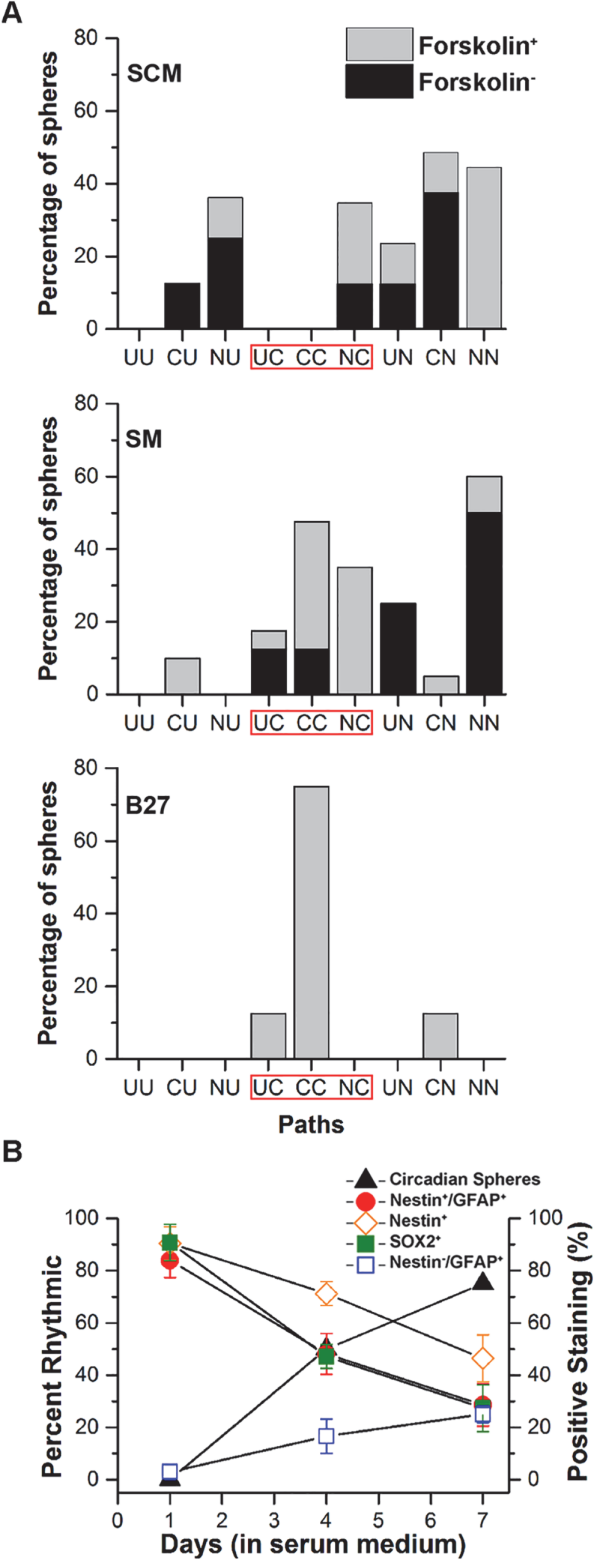
The rhythmic state of spheres during early and late exposure to three culture conditions. **A:** Spheres were maintained in either SCM, SM or B27 medium. Spheres were imaged immediately after a forskolin treatment to synchronize circadian clock cells or after no treatment. Shown is the percentage of spheres that began in a particular state (C: circadian, U: ultradian, N: nonrhythmic) during the first 3 days of imaging (early) and their state during the final 3–4 days (late) of imaging sessions. Under differentiating conditions (SM or B27) the three paths to the circadian state (UC, CC, and NC) were most commonly observed. **B:** The increase in the percentage of spheres showing circadian rhythms is correlated with an increase in the differentiation marker Nestin^-^/GFAP^+^ and negatively correlated with the decline in stem cell markers (Nestin^+^/GFAP^+^, Nestin^+^, and SOX2^+^) during 7 days in SM.

To represent the fate that spheres followed in the three different media conditions, spheres were grouped by their initial state during the first 3 days of imaging (early) and their state during the final 3–4 days (late). These categories consist of nine paths that spheres could take during differentiation and are shown in Fig 2A, in which “UU”, “CC”, and “NN” represent spheres that remained ultradian, circadian, or non-rhythmic throughout 5–7 days of imaging in SCM, SM, and B27. The most common path taken by neurospheres in SM or B27 was to the circadian state during the late stage of imaging.

### Stem cell state declines following transition into differentiation-inducing environments

**Fig 3:**
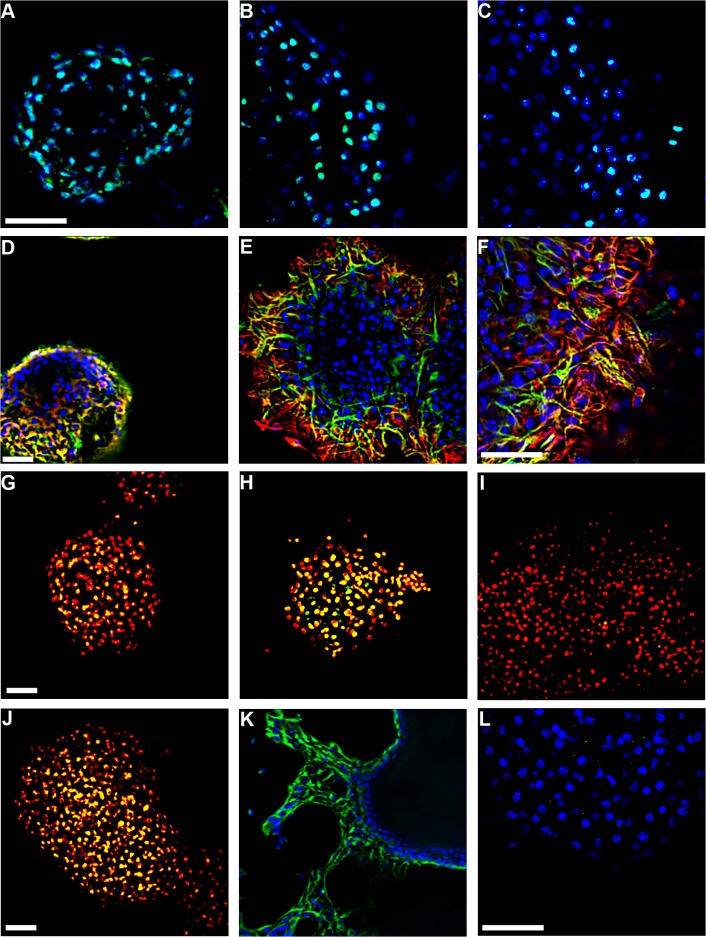
Emergence of circadian rhythms before fully differentiated neurons appear. Spheres were synchronized by forskolin treatment and fixed after differentiation in SM or B27 medium, mimicking BLI conditions. Hoechst (blue) or propidium iodide (red) were used as nuclear stains. NSPCs were identified as SOX2^+^ (cyan; **A**-**C**: after 1, 4, 7 days in SM), Nestin^+^/GFAP^+^ (yellow; **D**-**F**: after 1, 4, 7 days in SM; red: GFAP, green: Nestin) or Msi1^+^ (yellow; **H**: after 3 days in SM). Additional spheres were fixed after differentiation in medium with serum or B27 supplement to stain for progenitor cells as Dcx^+^ (Yellow; **G**, **J**: after 4 days in SM or B27, respectively). Immature neuronal cells were identified as BetaIII-tubulin^+^ (Green; **K**: after 5 days in B27 medium), and mature neuronal cells as NeuN^+^ (Yellow; **I**: after 4 days in SM and Green; **L**: after 4 days in B27 medium). Scale bars = 50 μm, and **A**-**C**, **E**, **H**, **I**, and **K** are at the same magnification.

Following immunofluorescence staining for markers of stem cells and differentiated cells, it was clear that the population of identified NSPCs declined as differentiation progressed, but undifferentiated cells remained throughout the 7 days of BLI (Fig 2B). The neurospheres did not fully differentiate into a complete monolayer cell culture during BLI. To characterize the extent of differentiation, partly differentiated cultures were fixed at different time intervals, after the 1st, 4th and 7th day of differentiation in SM or B27 medium, mimicking conditions during BLI. NSPCs within neurospheres were identified by immunofluorescence using anti-SOX2 [41] (Fig 3A–3C), anti-Nestin and anti-GFAP [42] (Nestin^+^/GFAP^+^, Fig 3D–3F), anti-Msi1 [42] (Fig 3H), and anti-Nestin alone (S1A–S1C Fig). Hoechst 3342 or propidium iodide (PI) were used to identify cell nuclei.

During neurogenesis in the SVZ, neuroblast (type C) cells that are positive for doublecortin (Dcx), a marker for the neuroblast-like cells, migrate through the RMS to the OB [43]. To determine whether neuroblast-like cells were present during BLI, neurospheres were immunostained for Dcx after 4 days in SM or B27 medium (Fig 3G and 3J, respectively). Dcx^+^ cells were significantly more abundant in neurospheres maintained in B27 medium (57.71 ±7.67%, 280, n = 7) when compared to SM (Table 2; t = 3.820, p<0.001). Mature neuronal cells were almost entirely absent when circadian rhythms were detected at the end of 4 days of differentiation in SM (Table 2, Fig 3I) or B27 medium (1.30 ±1.1%, n = 6, Fig 3L), as determined by staining against the marker for terminally differentiated neurons NeuN [44]. After four days of differentiation in B27 medium, neurospheres were positive for BetaIII-tubulin (41.40 ±7.1%, n = 7, Fig 3K), a marker for immature neurons [45]. No BetaIII-tubulin^+^ cells were observed in neurospheres after 4 days of differentiation in SM (S1D Fig). NeuN^+^ cells were present in neurospheres differentiating for 7–8 days in B27 (S1E Fig).

**Table 2:** Cell types identified by markers for stem cells and differentiated cells in SM.

**Cell type**	**Day 1**	**Day 4**	**Day 7**
SOX2^+^/Hoechst*	90 ±6.9% (119, n = 8)	47.11 ±4.6% (100, n = 9)	27.38 ±8.9% (110, n = 11)
Nestin^+^/GFAP^+^/Hoechst	83.90 ±6.4% (62, n = 7)	48.12 ±7.8% (297, n = 9)	25.59 ±8.1% (255, n = 11)
Nestin^+^/PI	90.44 ±6.4% (102, n = 10)	71.22 ±4.6% (182, n = 9)	46.40 ±8.9% (202, n = 7)
DCX^+^/PI	N.A.	35.95 ±13.3% (128, n = 9)	N.A.
BetaIII-tubulin^+^/PI	N.A.	1.28 ±1.1% (76, n = 7)	N.A.
NeuN^+^/PI	N.A.	1.96 ±2.6% (76, n = 7)	2.58 ±4.0% (61, n = 6)
Nestin^-^/GFAP^+^/Hoechst	2.94 ±2.7% (62, n = 7)	16.59 ±6.5% (297, n = 9)	25.04 ±5.1% (255, n = 11)

Neurospheres were maintained in SM for the number of days indicated. Shown are the percentages of cells in optical sections that were positive for cell markers or combinations of markers followed by standard deviation. In parentheses are the total number of cells in the section, identified by nuclear stains (Hoechst 3342, propidium iodide), and the number of spheres analyzed (n). N.A. = not available. Cells were imaged with a 20X objective lens, except 40X was used where indicated (*).

We determined the relationship between the stem cell states of the SVZ cultures in differentiating medium to their rhythmicity. The BLI time-series data in Fig 1 showing the percentage of rhythmic spheres was compared with the percentage of cells that were SOX2^+^, Nestin^+^/GFAP^+^, or Nestin^+^ alone (without co-localization). Cells that were GFAP^+^/Nestin^-^ (mature astroglia) were also quantified to characterize the differentiation state of the culture. Fig 2B shows that a negative correlation exists between circadian rhythmicity and stem cell state of the sphere cultures (SOX2: slope = -0.8496 ±0.02085, R^2^ = 0.9988, p = 0.0156; Nestin^+^/GFAP^+^: slope = -0.7801 ±0.01338, R^2^ = 0.9995, p = 0.0099; GFAP^+^/Nestin^-^: slope = 0.2915 ±0.01603, R^2^ = 0.9940, p = 0.0336). As shown, the percentage of spheres that were rhythmic correlated with the decrease in stemness and increase in differentiation status.

### Forskolin synchronizes circadian clocks within neurospheres

**Fig 4:**
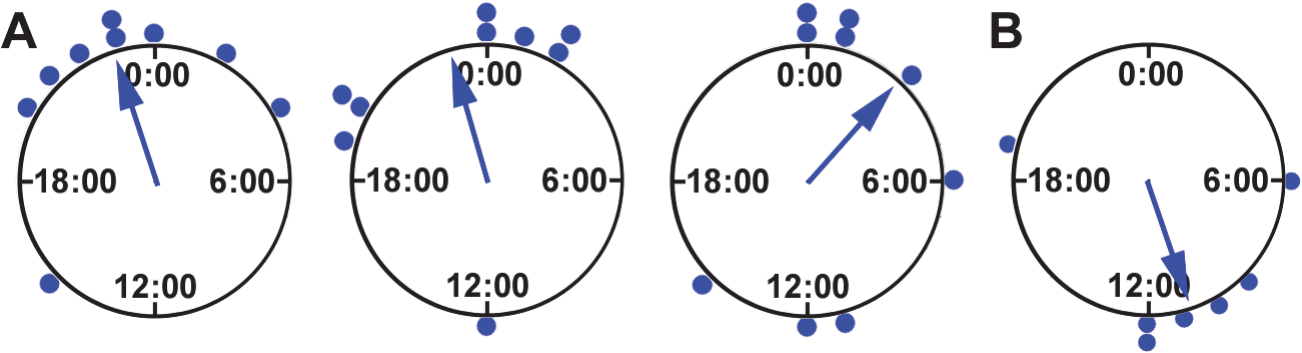
Synchronization of circadian neurospheres. **A:** Shown are the phases of individual neurospheres in SM expressing circadian rhythms plotted according to the first three peaks during imaging (left to right). Hour zero corresponds to the time when the forskolin treatment was removed and then projected over the next three days at 24-hour intervals. The mean vector of phases for individual spheres (arrow) had a significant magnitude (Rayleigh test, p<0.05) only during the first two cycles. **B:** Phases of spheres in B27 medium during the first circadian cycle and mean vector showing significant clustering.

Forskolin was used to synchronize clocks within spheres, but to verify that it was effective in these undifferentiated cultures we compared the phase at the first, second, and third peaks after the forskolin pulse for spheres expressing a circadian rhythm. In SM the 1^st^ and 2^nd^ peaks were clustered significantly near the predicted phase, approximately 24 hours after the treatment, according to the Rayleigh test (Fig 4A). The mean vector occurred at 22:44 ±3.45 hrs SD (Z = 3.98, p = 0.014) and 22:55 ±4.03 (Z = 2.96, p = 0.047) for the 1^st^ and 2^nd^ peaks, respectively, where 0:00 indicates the end of the 2-hr forskolin pulse. The spheres were not significantly clustered by the third peak (Z = 0.78, p = 0.471, n = 10 spheres for all peaks). The phases of spheres imaged in B27 medium were significantly clustered only during the first circadian cycle (Z = 2.939, p = 0.047, n = 7), and the mean vector was at 10:42 ±3.56 hrs, about 12 hours out-of-phase with the SM group (Fig 4B).

Although forskolin was used here to synchronize clock cells, it has been reported to have differentiation-inducing properties as discussed previously [39]. In one study, 5 μM forskolin in medium with 0.5% serum caused differentiation of mouse whole-brain neural stem cultures after 7 days of exposure [46]. To test whether the 2-hr forskolin pulse used here to provide synchronization between spheres and within spheres caused differentiation, the percentage of cells expressing the stem cell marker SOX2 was determined by immunofluorescence. There were no significant differences in the prevalence of SOX2^+^ cells when comparing forskolin-treated and untreated spheres after 96 hrs in SM (t = -1.59, p>0.12). The percentage of SOX2^+^ cells in 4-day SM spheres was 47.11% ±1.46 with forskolin treatment and 43.26% ±1.89 without treatment.

## Discussion

### Initiation of circadian rhythms during neurosphere differentiation

The circadian rhythms in *mPer1* gene expression observed in individual SVZ neurospheres indicate that spheres contain a functional circadian clock while they differentiate in vitro. As predicted, stem cell markers were identified throughout the neurosphere, suggesting that circadian rhythms originated within NSPCs. Similarly, tumorspheres that form in vitro from cancer stem cells are also enriched with stem cell markers and express circadian rhythms in *mPer2* activity [39]. Although some cells within the SVZ neurospheres may not contain a circadian clock, a substantial number of cells are rhythmic and are in an adequately close phase relationship with each other to provide a measurable ensemble circadian rhythm from entire spheres.

Neurospheres also displayed fast, ultradian oscillations of *mPer1* gene expression, particularly when maintained in SCM. This is the first time that ultradian or circadian rhythms in *Per1* gene expression have been described in neural stem cell cultures, and it suggests that media conditions alter both the differentiation and rhythms of these cells. It agrees in principle with previous studies of mouse embryonic stem cells in which differentiation was correlated with circadian rhythmicity, and dedifferentiation suppressed circadian rhythms [20]. It is possible that the observed ultradian oscillations in *mPer1* within SVZ neurospheres actually result from uncoupled cellular circadian oscillations that appear at the whole-neurosphere level as fast oscillations created by the multiple peaks of desynchronized rhythms. However, the adenylate cyclase activating agent forskolin was used to bring circadian oscillators into phase with each other. This treatment synchronizes circadian oscillators in rat-1 fibroblast cell cultures [47] and tumorsphere cultures [39] with the first peak of the circadian oscillation in *Per1* mRNA occurring about 20 hours after the treatment [47].

Switching neurospheres from SCM to either SM or B27 induced differentiation and increased the proportion of spheres expressing circadian rhythms. It is possible that this removal of EGF or FGF2 from the medium initiated emergence of circadian rhythms by allowing the cells to differentiate, suggesting that the more immature NSPCs are unable to generate circadian timing. There are two possible causes for this result: First, the necessary full set of core circadian clock genes are not yet expressed at this stage of differentiation. However, expression of the major core clock genes in mouse neurospheres has been reported [48]. Second, the clock genes are expressed, but the oscillator cannot operate because necessary non-rhythmic positive inputs are missing or an inhibitory factor is present in the spheres during early differentiation. It is also possible that the growth factors in SCM suppress functioning of the clock mechanism. It seems unlikely that either of the added growth factors can completely suppress circadian activity because circadian rhythms were detected in SCM, although these were rare during the late component of imaging sessions.

Stem cell state and circadian rhythmicity were negatively correlated, but the rhythmicity of spheres undergoing differentiation in vitro from the most stem-like state in SCM did take various paths, such as changing from a non-rhythmic or ultradian state to circadian. When examining all of the possible paths, the neurospheres that were ultimately circadian during the last 3–4 days of imaging were ones that had been given forskolin and then maintained in either SM or B27 medium. Spheres under these medium conditions attached and began propagating into neuroblast and glial-like cells that, by day 6 or 7, stained for Dcx and GFAP, respectively, further indicating that more differentiated spheres are more likely to be circadian.

### Origins of neurosphere circadian rhythms

It is likely that neurospheres are composed of many individual circadian oscillator cells as well as non-clock cells that are unable to sustain a circadian rhythm without input of timing information from other cells. Similarly, some brain areas when isolated as explant cultures produce circadian activity, whereas others do not. Several major brain structures have been grouped into three categories: endogenous circadian clocks, rapidly damping slave oscillators (producing only a single cycle without timing input), and non-circadian (lacking observable circadian rhythms) [49].

One reason why circadian rhythms were not common in SCM spheres could be because individual circadian clock cells are present but they are not adequately synchronized to a common phase to be detected in the whole-sphere recordings. To test for this possibility, spheres in SCM were given a pulse of forskolin before imaging but the percentage of circadian spheres did not increase. It is possible, but seemingly unlikely, that the less differentiated cells present in SCM are not responsive to forskolin but might contain a circadian clock. The circadian rhythms in spheres imaged in SM did respond to forskolin by showing a significantly clustered phase that was near the phase expected for this treatment, about 24 hours after the pulse [47]. By the third cycle, the forskolin-treated SM spheres had drifted out of phase and were no longer clustered significantly, according to circular statistics. Spheres in B27 medium given a forskolin pulse were significantly clustered, but this occurred at a phase 12 hours away from the expected phase. It is possible that the transition into B27 medium had its own phase-shifting effect that acted in combination with forskolin. B27 medium has been shown to elevate *mPer1* expression in cortical astrocyte cultures [50], suggesting that it could cause a phase-shift by altering the level of this core clock component.

Although the forskolin-treated SM spheres were in SCM during the forskolin treatment, and so were mostly undifferentiated, some circadian clock cells must have been present for the forskolin to produce synchronization. Because the forskolin-treated SCM and SM spheres were initially in the same state of differentiation but SCM spheres showed few circadian rhythms for the next several days, it is likely that the clock cells present were too scarce to be detected within the larger cell population of non-circadian cells. In the SM spheres, on the other hand, these early synchronized clock cells likely proliferated in the presence of serum while other cells also differentiated and proliferated. It is possible that differentiating non-clock NSPCs began to function as circadian oscillators and were synchronized to the early clock cells through the close interactions present in neurospheres (gap junctions, NCAMs, integrin, etc.) [51,52]. Similarly, circadian clock cells may be present at very low numbers during early stages of embryonic development before circadian rhythms can be detected [53]. Therefore, the data are more reasonably explained if a small number of clock cells are present in neurospheres in SCM and these maintain a common phase and temporal order during the differentiation process in SM and B27 medium, while remaining NSPCs differentiate into clock cells.

### Circadian rhythms in progenitor cells

As neurospheres differentiated in SM, the initial abundance of stem cell markers declined (SOX2, Nestin, GFAP^+^/Nestin^+^), cells with differentiation markers increased (Dcx, BetaIII-tubulin, NeuN and GFAP), and the percentage of circadian spheres increased. The present study was not designed to determine whether individual, identified stem cells express circadian rhythms. Nevertheless, results did indicate that progenitor cells are functional circadian clocks because of the lack of mature neurons in spheres after 3–4 days in SM even though 50% of the spheres were able to generate circadian rhythms. Bioluminescence images of spheres after 4 days in SM showed that the signal originated from cells throughout the spheres (S1F Fig). The astrocytes that were detected at this point in culture could have been responsible for generating the rhythms because astroglial circadian clocks have been described in vitro [54]. Whether the small minority of astrocytes present (16.5% according to GFAP^+^/Nestin^-^ staining) were able to drive circadian rhythms in a much larger population of progenitor cells is not known. However, glial cell secretions can alter activity of neural circadian cells in drosophila [55] and mice [56].

In a previous study, circadian rhythms were described in neural progenitor-like cells, but these were in a glioblastoma-derived cell line rather than the non-transformed primary cultures used here [57]. The present results are not in agreement with a previous study of circadian gene expression in SVZ cell cultures in which a circadian clock appeared first in mature cells, and no circadian rhythms in differentiating neurospheres were reported [48]. Similarly, rapidly differentiating cells lack a detectable circadian rhythm during mouse spermatogenesis [58].

The circadian rhythms observed in neurospheres maintained in B27 medium for 4 days that were predominantly positive for Dcx indicates that circadian rhythms originate in neural progenitor cells, particularly neuroblasts (S2 Fig), after their fate is determined to become interneurons (granule or periglomerular cells) in the OB [59,60]. It also suggests that circadian timing in neuroblasts may function during their migratory behavior in the RMS. We predict that neuroblasts become circadian granule cells upon final differentiation, although a previous study did not find circadian rhythms in the granule cell layer of the OB [15]. Nevertheless, circadian rhythms in mature olfactory granule cells may aid in discrimination between closely related odors, an important adaptive ability for which neural stem cells may be required [6], and may improve this sensation at times of day when that is most important [61]. Similarly, circadian rhythms in SVZ progenitor cells might serve in establishing the time of day when final neuronal differentiation occurs, optimizing availability of nascent cells with a lower threshold for the excitation needed to perform odor discrimination [6].

### Possible importance of *mPer1* in neurogenesis

It is clear that *mPer1* is expressed in spheres that are not showing circadian oscillations through BLI. A question that remains is whether *mPer1* in differentiating progenitor cells serves in the process of neurogenesis, similar to what has been observed for other clock genes. Studies described an increased expansion rate of neurospheres from *mPer2* ^*-/-*^ knockout mice that lack circadian rhythms [62]. Similarly, neural progenitor cell proliferation is increased in DG neurospheres from mPer2^brdm1^ mutant mice [63]. BMAL1 or CLOCK may also serve in neurogenesis, with or without a functioning circadian clock, as shown by RNA inhibition that decreased differentiation markers [48].

Along with a circadian function, *mPer1* gene expression may also have an important role when expressed in ultradian oscillations such as those observed in SCM neurospheres. These rhythms may be working with stem cell-maintaining genes such as the *hes* family that are expressed in ultradian oscillations during neurogenesis and embryogenesis where they play an important role in repressing genes used in differentiation [64]. Neurogenesis and circadian oscillators both rely on a collection of basic-helix-loop-helix (bHLH) transcription factors, some of which are shared between these two time-dependent processes. For example, one gene promoter element used in circadian transcriptional control (an alternative E-box) includes the N-box that binds the bHLH HES1 protein [65]. The circadian clock could also have a direct effect on differentiation through its control of an E-box element of the Pax6 gene promoter [66]. Pax6 serves in determining the rate and direction of neurogenesis in the OB [67–69].

If circadian timing, rather than non-rhythmic clock gene expression, has a functional role in adult NSPCs during early stages of differentiation, circadian oscillations may modulate particular differentiation events [48,62]. In a similar way, daily oscillations in brain cortisol appear to gate cell proliferation in adult mouse hippocampus [11]. Again, coupling between circadian and stem cell-maintaining genes could serve in this control [22]. Alternatively, neurogenesis and circadian timing processes could act independently within the same cells despite predicted interactions between the bHLH transcription factors acting on N-box and E-box elements. The SVZ neurosphere cultures examined here provide a useful assay to investigate the role of circadian clocks and clock-controlled genes in adult neurogenesis. Understanding the relationship between circadian clock genes and neurogenesis could provide new targets for more effective treatments and prevention of neurological disorders such as Parkinson’s and Alzheimer’s diseases that are suitable for stem cell therapies [70]. If circadian timing acts on differentiation, then circadian expression patterns may be manipulated to induce NSPCs to differentiate more readily into specific cell types needed to compensate for neural deficits.

## Conclusions

This exploration of the circadian timing abilities of NSPCs identified autonomous circadian oscillators that are visible when growth conditions induce differentiation. Circadian rhythms appear in neurospheres before mature neurons are present, indicating that NSPCs, which are very prominent in neurospheres, also have functional circadian clocks. The results neither confirm nor deny existence of circadian clocks in the most undifferentiated neural stem cells, the radial glia-like cells. When NSPCs of the SVZ are allowed to differentiate into neuroblast-like cells of the RMS they appear to have circadian properties that could be adaptive for their unique transit to become OB interneurons.

## Supporting Information

S1 Fig Images of spheres during differentiation. Nestin^+^ cells (green) at days 1 (**A**), 4 (**B**), and 7 (**C**) in SM with PI-stained nuclei (red). **D**: Lack of BetaIII-tubulin^+^ cells (green) with PI (red) in a neurosphere at day 4 in SM. **E**: NeuN^+^ cells (green) with Hoechst-stained nuclei (blue) at day 7 in B27 medium. Scale bars = 50 μm. **F**: Three neurospheres in SM used for measuring circadian rhythms in *mPer1* expression. Top: Brightfield image at day 0. Bottom: Corresponding bioluminescence image at day 4. Average maximum signal was 444 ADUs ±49.0 (SD). Each pixel represents 61 x 61 μm.
(TIF)

S2 Fig Circadian rhythms during neurogenesis. A summary diagram predicting that neural stem cells (radial glia-like cells) residing in the SVZ lack a functioning circadian clock but can exhibit high-frequency oscillations in clock gene expression (green). They further differentiate into neuroblasts and enter the RMS where they exhibit circadian oscillations in clock gene expression (red). These cells migrate to the OB and differentiate into granule cells and may contribute to previously described OB circadian rhythms (red) [15].
(TIF)
