# Supplementary figures and images for: Development of Circadian Oscillators in Neurosphere Cultures during Adult Neurogenesis
Source: PLoS One. 2015 Mar 31;10(3):e0122937. doi: 10.1371/journal.pone.0122937 (PMC4380296; doi:10.1371/journal.pone.0122937)

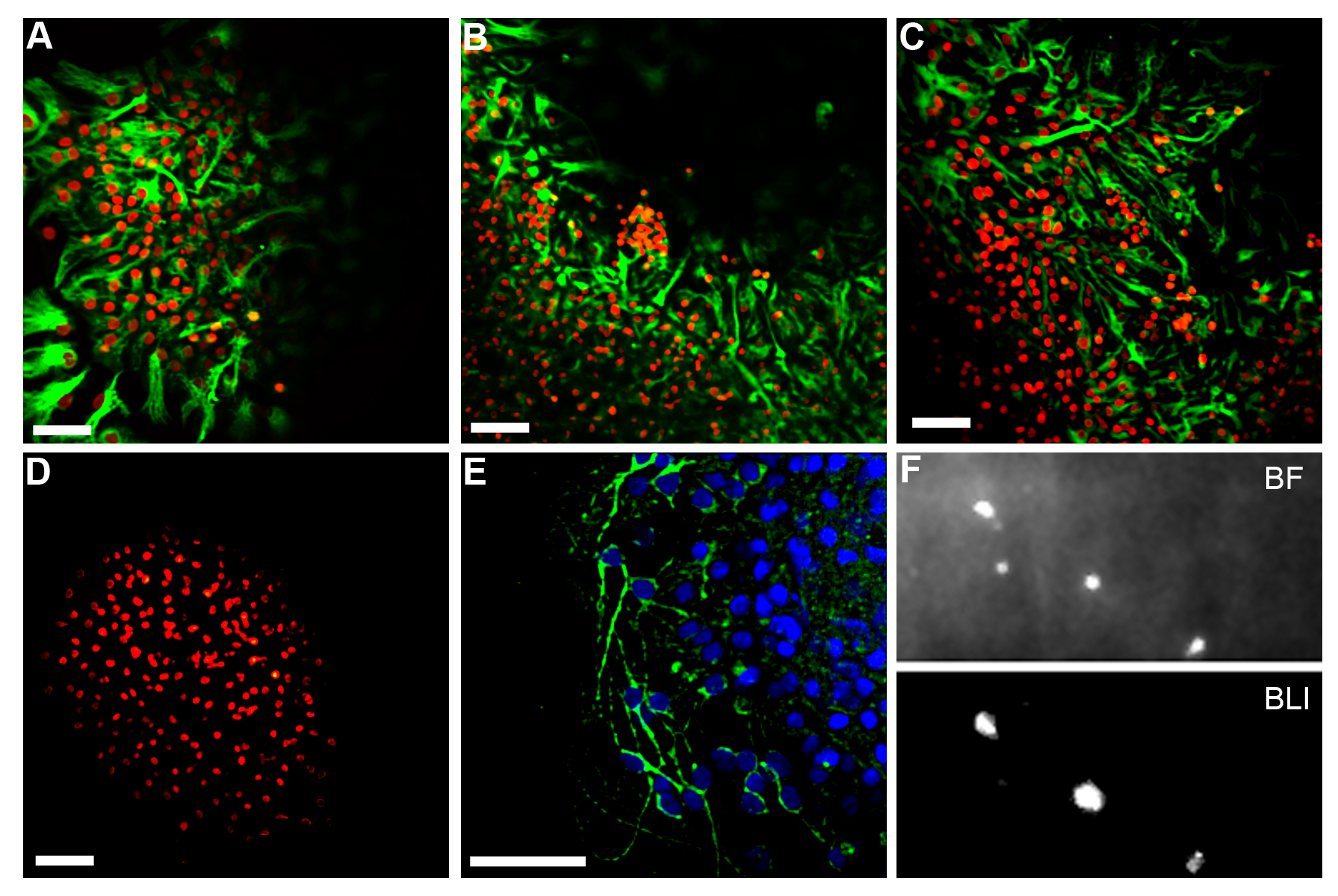

Supplement: S1 Fig — Nestin+ cells (green) at days 1 (A), 4 (B), and 7 (C) in SM with PI-stained nuclei (red). D: Lack of BetaIII-tubulin+ cells (green) with PI (red) in a neurosphere at day 4 in SM. E: NeuN+ cells (green) with Hoechst-stained nuclei (blue) at day 7 in B27 medium. Scale bars = 50 μm. F: Three neurospheres in SM used for measuring circadian rhythms in mPer1 expression. Top: Brightfield image at day 0. Bottom: Corresponding bioluminescence image at day 4. Average maximum signal was 444 ADUs ±49.0 (SD). Each pixel represents 61 x 61 μm. (TIF) [file pone.0122937.s001.tif]

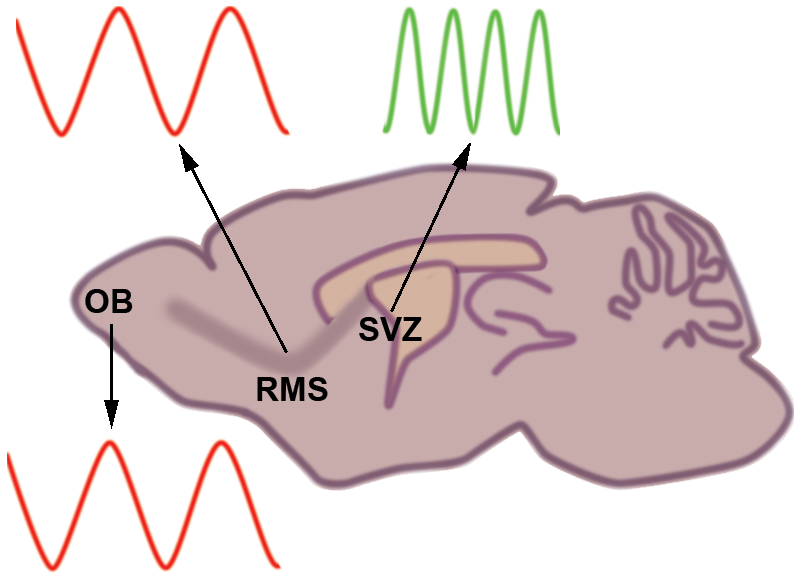

Supplement: S2 Fig — A summary diagram predicting that neural stem cells (radial glia-like cells) residing in the SVZ lack a functioning circadian clock but can exhibit high-frequency oscillations in clock gene expression (green). They further differentiate into neuroblasts and enter the RMS where they exhibit circadian oscillations in clock gene expression (red). These cells migrate to the OB and differentiate into granule cells and may contribute to previously described OB circadian rhythms (red) [15]. (TIF) [file pone.0122937.s002.tif]
